# Nipah Virus Infection in Dogs, Malaysia, 1999

**DOI:** 10.3201/eid1506.080453

**Published:** 2009-06

**Authors:** James N. Mills, Asiah N.M. Alim, Michel L. Bunning, Ong Bee Lee, Kent D. Wagoner, Brian R. Amman, Patrick C. Stockton, Thomas G. Ksiazek

**Affiliations:** Centers for Disease Control and Prevention, Atlanta, Georgia, USA (J.N. Mills, B.R. Amman, P.C. Stockton, T.G. Ksiazek); Regional Veterinary Diagnostic Laboratory, Petaling Jaya, Malaysia (A.N.M. Alim); Centers for Disease Control and Prevention, Fort Collins, Colorado, USA (M.L. Bunning); Office of the Surgeon General, Washington, DC, USA (M.L. Bunning); Department of Veterinary Services, Kuala Lumpur, Malaysia (O.B. Lee); Ithaca College, Ithaca, New York, USA (K.D. Wagoner)

**Keywords:** Dogs, Nipah virus, encephalitis, pigs, zoonoses, Malaysia, dispatch

## Abstract

The 1999 outbreak of Nipah virus encephalitis in humans and pigs in Peninsular Malaysia ended with the evacuation of humans and culling of pigs in the epidemic area. Serologic screening showed that, in the absence of infected pigs, dogs were not a secondary reservoir for Nipah virus.

During September 1998–April 1999, a viral disease associated with pigs resulted in at least 265 human cases of febrile encephalitis in Peninsular Malaysia; case-fatality ratio was 38% (*1*). The etiologic agent, Nipah virus (NiV; family *Paramyxoviridae,* genus *Henipavirus*), is believed to have entered pig populations in Perak state, central Malaysia, from a fruit-bat reservoir (*2*) before spreading by transport of pigs among farms. Bukit Pelanduk, Negeri Sembilan state, and adjoining Sepang, Selangor state, had the largest number of cases. The epidemic in that region was controlled by cessation of animal movement, destruction of pigs on affected farms, public education, use of personal protective equipment, and evacuation of humans from and quarantine of farms and villages within the epidemic area (*3*).

Although humans were most frequently infected after contact with live pigs (*4*–*6*), 8% of patients reported having had no direct contact with pigs, which suggests other sources of transmission to humans. One study reported an association with sick or dying dogs; case-patients were more likely than controls to report an increase in the number of sick or dying animals, including dogs (*5*).

During the outbreak, evidence of NiV infection was found in domestic animals such as goats and cats, but especially dogs (*2*). NiV infection was confirmed by immunohistochemical examination of 1 dead and 1 dying dog from the epidemic area. Both showed histologic evidence of severe disease (*7*). After pig populations were destroyed but before residents were allowed to return to their homes in the epidemic area, studies were undertaken to determine whether domestic animal populations maintained active infection in the absence of infected pigs. Dogs were especially suspected because they live commensally with both pigs and humans. Many dogs sampled from the Bukit Pelanduk and Sepang epidemic area around the time of the pig culling had antibodies to NiV or a Nipah-like virus (*2*). To test the hypothesis that NiV was being transmitted from dog to dog, we looked for evidence of the spread of infection among dogs outside the immediate disease-endemic area.

## The Study

The disease-epidemic zone in southwestern Malaysia was an area of small pig farms associated with a cluster of small towns in the states of Negeri Sembilan (Bukit Pelanduk, Sungai Nipah, Kampong Sawah) and adjacent Selangor (Sepang) (Figure). For 3 days (May 11–13, 1999), stray and pet dogs were sampled along 2 transects following major paved roads, through rural areas, leading from the periphery of the recognized disease-epidemic area (Figure). On days 1–3, samples were collected within 15–20, 8–15, and 0–8 km, respectively, of each transect.

**Figure:**
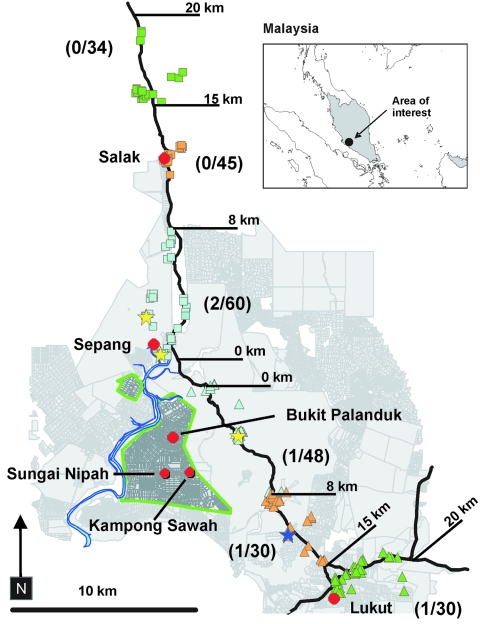
Sampling locations for 161 pet and 88 stray dogs along 2 transects that followed major roads leading north (squares) and southeast (triangles) from the Nipah virus encephalitis disease-epidemic areas in Bukit Pelanduk and Sepang, Malaysia. Color-coded squares and triangles refer to the transect interval where they were taken (light blue, 0–8 km; orange, 8–15 km; green, 15–20 km). The recognized disease-epidemic areas in pigs and humans are outlined by thick green lines. Sampling sites of Nipah virus antibody–positive dogs are indicated by stars (yellow, pet; blue, stray). Numbers in parentheses beside each transect indicate number antibody positive/number tested from each transect. Distances are in road kilometers; however, all antibody-positive animals were sampled within 5-km linear distances from the epidemic area.

Sampling followed 2 methods. For household pets, blood samples were collected from the dogs, geographic coordinates of the house were measured by using a geographic positioning system, and owners were asked about the animal’s potential exposure history (e.g., where it was kept, whether it was allowed to roam, health, diet, any presence on a pig farm, and if it had been sick during the previous 12 months). Free-roaming stray dogs without collars were killed by animal control personnel following routine protocols for rabies control and returned to a field laboratory in Bukit Pelanduk for sampling. Geographic coordinates and form-directed data (sex, age, apparent health) were recorded for each stray. A veterinary team collected blood and tissue samples (spleen, kidney, liver, lung) from each animal.

For comparison, blood samples from 109 dogs from the Kuala Lumpur area (29 from veterinary clinics, 19 from pounds, and 61 stray dogs) were collected. Hendra virus (HeV) is highly cross-reactive with NiV and was successfully used for initial screening in humans and animals during the outbreak (*8*). Samples were tested by using an indirect immunoglobulin (Ig) G ELISA with HeV antigen as described (*9*).

During the 3 days, 249 dogs were sampled; 161 blood samples were from pets, and 88 blood and tissue samples were from stray dogs. The daily number of samples increased along both transects as the study progressed, reflecting the improved efficiency of sampling teams (Figure).

Of the 249 blood samples, 4 (1.6%; 2 from each transect) had detectable antibodies reactive with HeV. The 56 blood samples from animals 15–20 km from the epidemic area had no detectable antibodies. There was 1 antibody-positive animal in the 8–15 km zone and 3 in the 0–8-km zone (Figure). Of the 4 antibody-positive dogs, 3 were pets from the 0–8-km zone. The antibody-positive stray was from the 8–15-km zone. The 109 blood samples from pet and stray dogs in Kuala Lumpur were HeV antibody negative.

## Conclusions

The finding that all 109 dogs from Kuala Lumpur (including at least 29 vaccinated pets) were antibody negative indicated that the immunoassay did not detect antibody elicited by common canine vaccines (canine distemper, hepatitis [adenovirus type II], parainfluenza, leptospira, and canine parvovirus) or other common paramyxovirus infections. The absence of NiV antibody in dogs >15 km from the epidemic area and the low prevalence in populations nearer the epidemic area provides evidence that the virus was not spreading by dog-to-dog transmission and that the dog population was not acting as an amplifying reservoir for NiV in the absence of infected pigs. In addition, there were no reports of unusual numbers of dead or sick dogs outside the immediate disease-endemic area.

Infected pigs in the area of the epidemic were destroyed March 1–April 16, 1999. Prevalence of NiV antibody in 63 dogs from within this area April 3–14, 1999, was 57%. Prevalence in 19 dogs from the same area April 23–May 4, 1999, was 26% (unpub. data). Although this reduction in prevalence is not significant (p = 0.23, 2-tailed Fisher exact test), it suggests that the virus was not being rapidly transmitted among dog populations after destruction of the pigs. Some of the animals that had been exposed to infected pigs died or were killed and were probably replaced by uninfected immigrant dogs. Alternatively, infection in the dog population may have been local and patchy, and the apparent temporal differences in prevalence may reflect geographic sampling bias. Dogs sampled during the earliest study period were taken by patrolling animal control personnel. The exact locations of sampling are unknown, but they were areas abandoned by humans.

The greater number of infected pets than strays reflects their greater representation in the sampled population. Antibody prevalences (3/161 [1.9%] for pets vs. 1/88 [1.1%] for strays) did not differ significantly (p = 1.00, 2-tailed Fisher exact test).

Of the 3 antibody-positive pet dogs, none was reported to have been sick within the past year, 2 were “always” allowed to roam free, and the third was “rarely” allowed to roam free. However, the owners of the third dog reported feeding it “pig bones.” We believe that these animals could have become infected through direct contact with infected pigs or by eating uncooked pork products. Some animals classified as strays may have been pets that were allowed to roam free without collars.

We cannot exclude the possibility that dogs may have remained infectious for some period after infection or that other dogs or even humans may have become infected through contact with infected dogs. However, our results indicate that such infection was rare and was insufficient to maintain and spread NiV in dog populations in the absence of infected pigs.
